# Factors Associated With Prehospital Delay Among Older Adults Surviving Ischemic Stroke: A Cross‐Sectional Study

**DOI:** 10.1002/brb3.71717

**Published:** 2026-08-20

**Authors:** Harit Sianghwong, Wipratchaya Thedthong, Pornchai Jullamate, Chanandchidadussadee Toonsiri, Wanporn Worapornpong

**Affiliations:** ^1^ Principal Investigator Queen's University Belfast, School of Nursing and Midwifery UK; ^2^ Faculty of Nursing Burapha University, Mueang Chonburi Chon Buri Thailand

**Keywords:** health perception, ischemic stroke, older adults, prehospital delay, social support, stroke knowledge

## Abstract

**Background:**

Ischemic stroke is a major public health concern and a leading cause of disability among older adults. Because treatment effectiveness is highly time‐dependent, timely medical intervention is crucial for improving outcomes, while delays in hospital arrival often result in poorer recovery and higher mortality.

**Aim:**

To examine prehospital delay and associated factors among older adults with ischemic stroke.

**Design:**

A Cross‐sectional study.

**Methods:**

This research was conducted with 106 older adults aged 60 years and above who survived an ischemic stroke and received care at Ban Phaeo Hospital, Thailand. Data were collected through structured interviews and medical record reviews between February and May 2025. Four variables were assessed based on theoretical and empirical rationale: stroke severity, stroke knowledge, social support, and perceived health status. Pearson's correlation analysis was used to assess bivariate relationships between these factors and prehospital delay. Multiple linear regression was subsequently performed to evaluate the independent association of each factor while adjusting for the others.

**Results:**

The mean prehospital delay was 253.36 min (SD = 278.70). Although this value falls near the upper boundary of the 4.5‐h (270‐min) therapeutic window, when mandatory in‐hospital processing times are considered, intravenous thrombolysis would be effectively precluded for most patients arriving at this stage. Prehospital delay was significantly and negatively correlated with stroke severity (*r* = −0.521, *p* < 0.001), stroke knowledge (*r* = −0.452, *p* < 0.001), social support (*r* = −0.468, *p* < 0.001), and perceived health status (*r* = −0.489, *p* < 0.001).

**Conclusion:**

This study found that stroke severity, stroke knowledge, social support, and perceived health status were significantly associated with prehospital delay among older adults. This study addressed a gap in stroke care literature by focusing on these factors in a low‐to‐middle‐income setting. It offers insights for healthcare professionals to design targeted interventions that may promote early treatment‐seeking behaviors and improve stroke outcomes.

**Impact and implication:**

These findings underscore the need for nurse‐led educational and community outreach programs to enhance symptom recognition, foster proactive health behaviors, and mobilize social support systems among older adults.

**Patient or public contribution:**

This research, patients only participated in the data collection process by completing the survey.

## 1 Introduction and Background

Ischemic stroke is a leading cause of death and long‐term disability worldwide, exerting a profound burden on individuals, families, and healthcare systems. Older adults are particularly vulnerable to its devastating effects, as age‐related physiological changes, comorbidities, and functional decline compound the risk of adverse outcomes. In Thailand, the public health significance of stroke is especially notable, with stroke ranked as the second leading cause of premature mortality (Institute for Health Metrics and Evaluation [IHME], 2021). This alarming statistic underscores the urgent need to address the factors associated with timely stroke management among the aging population.

Prompt medical intervention, particularly the administration of thrombolytic therapy within the 4.5‐h therapeutic window, has been shown to markedly reduce complications and improve neurological recovery (Powers et al., 2019). However, a persistent obstacle in achieving optimal outcomes is prehospital delay, defined as the time interval between symptom onset and hospital arrival. Previous studies have identified multiple factors influencing prehospital delay, including clinical severity, symptom interpretation, health literacy, social support, and perceived health status. For example, Faiz et al. (2019) demonstrated that lower stroke severity and limited symptom recognition were associated with prolonged delays in hospital arrival. Similarly, Kim et al. (2011) reported that inadequate awareness of stroke warning signs significantly increased prehospital delay among ischemic stroke patients. In low‐ and middle‐income settings, additional barriers such as limited access to emergency medical services (EMSs) and reliance on family decision‐making have further contributed to delayed treatment (Teuschl & Brainin, 2010; Pongpanich et al., 2020).

Older adults are particularly susceptible to prolonged prehospital delay due to age‐related cognitive decline, multimorbidity, functional limitations, and psychosocial barriers (Sungkarat et al., 2017). Misinterpretation of stroke symptoms as benign or attributable to normal aging often results in critical loss of time, further reducing eligibility for effective interventions. Despite ongoing health promotion campaigns and the expansion of EMSs in Thailand, a substantial proportion of stroke patients continue to arrive at hospitals beyond the therapeutic window for thrombolytic therapy, indicating persistent gaps in timely treatment‐seeking behavior.

Although previous studies have explored factors influencing prehospital delay, many have focused on mixed‐age populations or hospital‐level system barriers. Far fewer have examined this issue specifically among older adults, who may face unique challenges such as diminished cognitive function, multiple comorbidities, dependency on caregivers, and social isolation. Understanding the interplay of stroke knowledge, perceived severity, social support, and perceived health status within this vulnerable group is therefore critical for designing interventions that are both effective and contextually appropriate.

Furthermore, the Thai cultural context, in which family members and community caregivers play a central role in healthcare decision‐making, provides an important lens for examining the influence of social support on timely hospital arrival. Focusing on older adults in a middle‐income country, this study not only contributes to locally relevant evidence but also addresses a broader gap in global stroke care research, where low‐ and middle‐income settings remain underrepresented. Thus, this study is justified by its potential to generate actionable insights for gerontological nursing, community health practice, and national stroke policies aimed at reducing prehospital delay and improving outcomes among older stroke survivors.

An important conceptual distinction informing this study is the difference between stroke awareness and stroke action awareness. Stroke awareness refers to the recognition of stroke symptoms, whereas stroke action awareness encompasses the knowledge and motivation to immediately activate EMSs upon symptom recognition. Recent literature emphasizes that recognition alone is insufficient unless it translates into prompt emergency response (Zhao & Liu, 2017; Liu et al., 2025).

Several culturally adapted stroke action frameworks have been developed internationally to bridge this gap by linking symptom recognition directly to the local emergency number. The Stroke 1‐2‐0 strategy, developed for Chinese‐speaking populations and utilizing the Chinese emergency number 120 as a mnemonic, was introduced in The Lancet Neurology as a model for culturally relevant stroke action education (Zhao & Liu, 2017).

Similarly, Stroke 911 was developed for regions using 911 as the emergency number, including Spanish‐speaking populations in Mexico and the Americas, linking symptom recognition to emergency activation (Liu et al., 2022). A randomized comparison in Spanish‐speaking adults further demonstrated that action‐oriented strategies significantly outperform awareness‐only approaches in improving rapid emergency response (Marquez‐Romero et al., 2025). These frameworks underscore the importance of shifting from stroke awareness education to stroke action education, a principle directly relevant to the Thai setting where the emergency number is 1669.

### 1.1 Study Objectives

The overarching objective of this study was to examine factors associated with prehospital delay among older adults who survived an ischemic stroke in Thailand. Specifically, the study aimed to: 1) describe the demographic and clinical characteristics of older adults with ischemic stroke, 2) examine the average prehospital delay time among older adult stroke survivors, 3) assess the levels of stroke knowledge, perceived health status, and social support in this population, 4) determine the relationship between stroke severity, stroke knowledge, social support, perceived health status, and prehospital delay, and 5) explore subgroup differences in prehospital delay according to demographic and social characteristics (e.g., education level, living arrangement).

### 1.2 Aim and Research Question

This study aimed to examine prehospital delay and the factors associated with it among older adults with ischemic stroke.

## 2 Methodology

### 2.1 Research Design and Sampling

This study employed a cross‐sectional correlational design to examine the factors associated with prehospital delay among older adult survivors of ischemic stroke. Participants were recruited from the Outpatient Internal Medicine Department of Ban Phaeo Hospital, Samut Sakhon Province, Thailand.

Ban Phaeo Hospital is a secondary‐level public hospital serving a predominantly rural and semi‐urban population in Samut Sakhon Province, Thailand. Stroke care is delivered through an organized referral system, where suspected stroke cases are initially assessed in the emergency department and subsequently managed by internal medicine and neurology teams. Thrombolytic therapy is available during twenty‐four hours, with after‐hours cases requiring referral to tertiary hospitals (mechanical thrombectomy). EMSs are accessible; however, many older adults rely on family members rather than ambulance services for hospital transportation, which may contribute to treatment delays.

### 2.2 Eligibility Criteria

Participants were eligible for inclusion if they were aged 60 years and above, had a confirmed diagnosis of ischemic stroke, were fully conscious, and were able to communicate in Thai. Exclusion criteria included a diagnosis of cognitive impairment or psychiatric illness.

The age cutoff of 60 years was applied in accordance with Thailand's national definition of older adults and aligns with gerontological and public health research standards in Southeast Asia. This threshold reflects age‐related physiological changes, increased stroke risk, and distinct care‐seeking behaviors observed in older populations, thereby ensuring the relevance of findings to geriatric stroke care planning.

### 2.3 Study Setting and Sampling

A simple random sampling strategy was used to select participants from the stroke registry of Banphaeo Hospital between February and May 2025. The final sample size was calculated using G*Power software with a medium effect size of 0.24 based on previous research (Jinnattha Kamsarirak and Chanokporn Chitpanya, 2015)*, α =* 0.05, and power = 0.80, resulting in a required minimum sample of 106 participants.

### 2.4 Data Sources and Measurement

The selection of the four study variables (stroke severity, stroke knowledge, social support, and perceived health status). was guided by Diefenbach and Leventhal (1996). Common‐Sense Model of Illness Representation and by consistent evidence in prior literature identifying these factors as significant correlates of prehospital delay (Faiz et al., 2019; Kim et al., 2011; Teuschl & Brainin, 2010). Stroke severity was included as the primary clinical indicator of perceived urgency; stroke knowledge as the cognitive determinant of symptom recognition and action‐taking; social support as the socio‐cultural facilitator of emergency help‐seeking; and perceived health status as the individual appraisal of vulnerability. Together, these factors represent a comprehensive set of modifiable and theoretically grounded influences on prehospital delay.

Data were collected from two primary sources: structured interviews with participants using validated questionnaires and medical record reviews to confirm clinical characteristics and stroke diagnosis. These tools were either developed or adapted from validated instruments and were employed to collect comprehensive data relevant to the study objectives.

First, the demographics and clinical data: age, gender, education, marital status, occupation, comorbidities, and symptoms at onset were extracted from medical records.

Second, stroke severity was evaluated by physicians using a clinical assessment tool validated in Thailand using the National Institutes of Health Stroke Scale (NIHSS), developed by the Neurological Institute of Thailand in 2019 (Cronbach's *α* = 0.90), scores: 0–4 = mild, 5–14 = moderate, 15–42 = severe.

Third, the cognitive function screening test utilized the six‐item cognitive impairment test, translated and adapted by Sattawat Udonchart et al. (2018) from the original test by Brooke and Bullock (1999), which ensured participants’ ability to respond reliably and showed a Cronbach's alpha coefficient of 0.80. The scores were interpreted as: 0–7 = no cognitive impairment, 8–9 = mild impairment, and 10–28 = severe impairment.

Fourth, the stroke knowledge that focused on risk factors and warning signs of stroke consisted of a 12‐item instrument developed by the Region 10 of the Ministry of Public Health, Thailand. The tool was tested with 120 stroke patients and showed a Cronbach's alpha coefficient of 0.80. Its scores were categorized as low = (< 5), moderate = (5–9), and high = (> 9).

Fifth, social support was measured using the scale developed by Chantana Lortajakul (2006), translated from the ENRICHD Social Support Instrument. The scale includes 7 items. The total scores range from 0 to 24 and are interpreted as follows 17–24 indicates high support, 9–16 moderate support, and 0–8 low support. The reliability test yielded a Cronbach's alpha coefficient of 0.88 from 30 participants.

Sixth, the perceived health status was evaluated using a 10‐item Likert scale developed by Nanwalai Chaisawat and Sathit Jindavong (2017), which was adapted for older Thai adults from the original tool. The total score ranges from 10 to 50 and is categorized as follows: 36.68–50 = high perceived health, 23.34–36.67 = moderate perceived health, and 10–23.33 = low perceived health. The tool was tested with 30 older adult ischemic stroke patients and yielded a Cronbach's alpha coefficient of 0.92.

### 2.5 Conceptual Framework

This study developed its conceptual framework based on Leventhal et al. (1996) Common‐Sense Model of Illness Representation (CSM) and an integrative review of relevant literature. The CSM posits that individuals interpret and respond to health threats through cognitive and emotional representations of illness, which subsequently guide their decision‐making and health management behaviors. In the context of ischemic stroke, these representations critically shape whether and how quickly individuals seek medical care following symptom onset.

The framework incorporates two primary dimensions: the socio‐cultural context and individual illness representation. Within the socio‐cultural domain, social support reflects the influence of family, caregivers, and community networks on health decision‐making. In Thailand, where family structures play a central role in healthcare navigation, social support is expected to strongly affect the timeliness of seeking care.

The individual illness representation dimension captures perceptions of both health and illness threats. Perceived health status represents an individual's overall appraisal of well‐being, which can either motivate or delay care‐seeking in response to acute symptoms. Stroke severity, as the clinical manifestation of the illness, influences how individuals interpret urgency: severe or disabling symptoms are more likely to trigger rapid response, whereas mild or ambiguous symptoms may be dismissed. Additionally, stroke knowledge was incorporated as a cognitive determinant, reflecting an individual's awareness of warning signs and recommended actions, which has been consistently linked to reduced prehospital delay in prior studies (Faiz et al., 2019; Kim et al., 2011).

**FIGURE 1:**
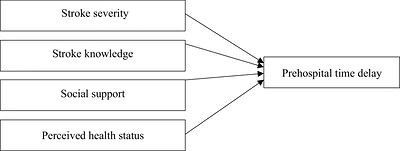
A diagram depicting the factors related to prehospital time delay among older adult stroke survivors.

Together, these four factors‐social support, perceived health status, stroke severity, and stroke knowledge‐are hypothesized to be associated with prehospital delay, defined as the time between symptom onset and hospital arrival. As illustrated in Figure 1, the framework highlights the interplay between socio‐cultural and individual‐level factors in shaping health‐seeking behavior. This model provides a theoretical and empirical basis for designing interventions aimed at reducing treatment delays and improving stroke outcomes among older adults.

### 2.6 Data Analysis

Data were analyzed using the SPSS software version 27. Descriptive statistics, including means, standard deviations, and frequencies, were utilized to summarize participants’ demographic characteristics. Pearson's product‐moment correlation coefficient was employed to assess the relationships between prehospital delay and the selected variables. A significance level of 0.05 was used to determine statistical significance. The dataset was screened for missing values. Fewer than 5% of cases had incomplete data, which were excluded listwise. No imputation was performed.

All final results presented in this manuscript are based on the complete dataset. As a sensitivity analysis, correlation and regression analyses were repeated after excluding extreme outliers (> 2 SD above the mean prehospital delay). The associations remained consistent in direction and significance, confirming the robustness of the presented findings. Detailed outlier‐excluded results are available upon request.

Assumptions for multiple linear regression, including normality, linearity, homoscedasticity, and multicollinearity, were evaluated prior to analysis. Variance inflation factor (VIF) values below 5 indicated no significant multicollinearity among predictor variables. Scatter plots, histograms, and Q‐Q plots confirming these assumptions are provided in the Supplementary Material.

### 2.7 Ethical Consideration

To protect human rights before conducting this study, the researcher informed the participants about the objectives of the study to make it clear that, in case they felt uncomfortable participating in the survey, they could withdraw their participation anytime without consequences of any kind or loss of benefits. The participants were given informed consent, for which they obtained information about the study's nature, as well as its benefits and consequences. Confidentiality was maintained by anonymizing data with coded identifiers and securely storing records. This study obtained ethical approval from the Human Research Ethics Board of Ban Phaeo Hospital (public organization) CoA No. 015/68, BGH REC No. 013/67, dated February 28, 2025.

## 3 Results and Findings

A total of 106 older adults with ischemic stroke were screened for cognitive impairment. The final sample consisted of 106 participants included in the analysis.

As shown in Table 1, the majority of the participants were aged between 60 and 69 years (53.77%), with an overall age range of 60 to 75 years. The mean age was 68.18 years (SD = 4.59). Males represented 62.57% of the sample. Approximately 60% had completed primary education, while 35.48% were self‐employed. A significant portion of the participants (96.54%) had comorbid conditions, with hypertension being the most common (90.56%). Furthermore, 75.43% were classified as having moderate stroke severity, with an average NIHSS score of 7.26 (SD = 5.71).

**TABLE 1:** Demographic and clinical characteristics of participants (*n* = 106).

Characteristics	N	%
Age (year) 60–69 70–75 (*M =* 68.18, SD *=* 4.59, Min = 60, Max = 75) Gender Male Female Educational Did not attend school Primary school Secondary school Diploma certificate Bachelor's degree Status Single Married Divorced Occupation Unemployed Business owner Self‐employed Agriculturist Retired from government Underlying factors Hypertension Heart disease Diabetes mellitus Dyslipidemia Kidney disease Severity of ischemic stroke (NIHSS) Normal (0) Mild (1–4) Moderate (5–14) Severe (15–24) (*M =* 7.26, SD *=* 5.71, Min = 0, Max = 16) Symptoms Numbness Weakness Dysarthria Ataxia Cognitive impairment No	57 49 66 40 2 64 20 17 3 5 85 16 10 11 30 35 20 96 18 72 67 17 8 45 29 24 75 99 66 6 100	53.77 46.23 62.57 37.43 1.90 60.00 19.24 16.00 2.86 4.71 80.18 15.11 9.43 10.37 28.30 33.01 18.86 90.56 16.98 67.92 63.20 16.03 7.55 42.45 27.36 22.64 70.75 93.40 62.27 5.66 100

### 3.1 Descriptive Results of Psychosocial Variables

Table 2A presents the descriptive statistics for the four psychosocial study variables. The mean stroke knowledge score was 6.83 (SD = 2.14; Range 2–12), indicating a moderate level of knowledge among participants, with the majority demonstrating limited awareness of early warning signs. This corresponds to the moderate category (Scores 5–9) of the 12‐item instrument. The mean social support score was 14.27 (SD = 4.51; Range 4–24), predominantly in the moderate range (Scores 9–16), reflecting reliance on family caregivers rather than broader community networks. The mean perceived health status score was 28.64 (SD = 6.73; Range 10–45), also rated as moderate (Scores 23.34–36.67), with many participants reporting chronic health conditions that influenced their self‐assessment. The mean NIHSS score was 7.26 (SD = 5.71; Range 0–16), classifying the majority of participants in the moderate severity category. These descriptive values, combined with the observed correlations, indicate that lower levels of stroke knowledge, social support, and perceived health status were each associated with longer prehospital delays. Table 2B

**TABLE 2a:** Descriptive statistics of psychosocial variables and prehospital delay.

Variable	Mean	SD	Range	Interpretation
Stroke knowledge	6.83	2.14	2‐12	Moderate
Social support	14.27	4.51	4‐24	Moderate
Perceived health status	28.64	6.73	10‐45	Moderate
Stroke severity (NIHSS)	7.26	5.71	0‐16	Moderate
Prehospital delay (minutes)	253.36	278.70	—	Near upper limit of therapeutic window

*Note*: The correlation coefficients analysis presented in Table 2B demonstrated that prehospital delay was moderately and negatively correlated with stroke severity *(r = –0.521, p < 0.001)*, stroke knowledge (*r =* –0.452*, p <* 0.001), social support (*r =* –0.468, *p <* 0.001), and perceived health status (*r = –*0.489, *p <* 0.001).

**TABLE 2b:** Pearson correlation matrix among prehospital delay and predictor variables (*n =* 106).

Variable	1. PD	2. SS	3. SK	4. Sup	5. PHS
1. Prehospital delay	1				
2. Stroke severity	−0.521***	1			
3. Stroke knowledge	−0.452***	0.302**	1		
4. Social support	−0.468***	0.273**	0.454***	1	
5. Perceived health status	−0.489***	0.309**	0.389***	0.487***	1

Abbreviations: PD = prehospital delay, PHS = perceived health status, SK = stroke knowledge, SS = stroke severity, Sup = social support.

***p < 0.001; **p < 0.01 (two‐tailed).

*Note*: Diagonal values (shaded) represent perfect self‐correlations. Negative correlations with prehospital delay indicate that higher scores on each predictor variable are associated with shorter delays.

*Note*: Negative correlations indicate that higher scores in each variable are associated with shorter prehospital delays.

The average time from the onset of stroke warning symptoms to hospital arrival was 253.36 min (SD *=* 278.70). Although this value technically falls near the upper boundary of the 4.5‐h (270‐min) therapeutic window, a hospital arrival time of 253 min effectively precludes intravenous thrombolysis in practice. When mandatory in‐hospital door‐to‐needle processing time (including emergency triage, neuroimaging, and laboratory evaluation, typically 30–60 min) is accounted for, patients arriving at this stage cannot receive IV tPA within the treatment window.

This study sought to examine factors associated with prehospital delay among older adults with ischemic stroke, focusing on the relationships between stroke severity, stroke knowledge, social support, perceived health status, and treatment‐seeking time. The findings revealed that longer prehospital delay was significantly associated with lower stroke severity, limited stroke knowledge, lower levels of social support, and poorer perceived health status. These results align with prior research indicating that both clinical and psychosocial factors play a central role in shaping response behaviors to stroke symptoms (Faiz et al., 2019; Kim et al., 2011).

Exploratory analyses indicated that participants with higher educational attainment (secondary level or above) experienced significantly shorter prehospital delays compared to those with primary education or no formal schooling. Older adults living with family members also arrived at the hospital sooner than those living alone. No statistically significant gender differences in prehospital delay were observed. These findings suggest that educational background and living arrangements may partially explain variability in delay predictor variables, although larger studies are needed to confirm these associations.

Multiple linear regression analysis to further examine the independent associations of the studied variables with prehospital delay, a multiple linear regression analysis was performed. The model was statistically significant (*F* = 35.12, *p* < 0.001), explaining 56% of the variance in prehospital delay (adjusted *R* ^2^ = 0.56). As shown in Table 3, all four variables remained significantly associated with prehospital delay in the multivariable model. The 95% confidence intervals for each regression coefficient are presented in Table 3.

**TABLE 3:** Multiple linear regression analysis of factors predicting prehospital delay (*n =* 106).

Predictor variable	b	SE(*b*)	𝛽	95% CI	VIF	*t*	*p*‐value
Stroke severity	−25.12	4.20	−0.32	−33.45 to −16.79	1.42	−4.12	< 0.001
Perceived health status	−15.60	3.50	−0.24	−22.53 to −8.67	1.51	−3.05	< 0.001
Social support	−10.80	2.90	−0.19	−16.53 to −5.07	1.33	−2.48	< 0.001
Stroke knowledge	−12.45	3.15	−0.17	−18.67 to −6.23	1.47	−2.21	< 0.001
(Constant)	520.45	45.30	—	430.96 to 609.94	—	11.49	< 0.001

*Note*: *R^2^ =* 0.58, adjusted *R^2^ =* 0.56, *F =* 35.12, *p <* 0.001, VIF = variance inflation factor.

*Note*: (95% confidence intervals): stroke severity: 95% CI [−33.45, −16.79]; perceived health status: 95% CI [−22.53, −8.67]; social support: 95% CI [−16.53, −5.07]; stroke knowledge: 95% CI [−18.67, −6.23]; constant: 95% CI [430.96, 609.94]. *b* = unstandardized regression coefficient; SE(*b*) = standard error; β = standardized coefficient.

## 4 Discussions

This study identified key psychosocial and clinical factors associated with prehospital delay among older adults with ischemic stroke. The mean prehospital delay of more than four hours represents a substantial barrier to timely access to thrombolytic therapy and limits opportunities for optimal neurological recovery.

Comparisons with recent studies from low‐ and middle‐income countries (LMICs) demonstrate that prolonged prehospital delay is a persistent and shared challenge across diverse resource‐limited settings. In Brazil, the implementation of a state‐wide telemedicine stroke network in Piauí reported a mean onset‐to‐door time of approximately 3.3 h, with thrombolysis administered to only a subset of eligible patients, underscoring the continuing impact of system‐level constraints despite organizational innovations (Ricarte et al., 2025).

In Senegal, prehospital delays frequently exceeded recommended treatment windows, with substantial delays observed in patient transfer to higher‐level facilities capable of providing acute stroke care (Damon et al., 2022). Similarly, an audit study from Mozambique reported a median total delay of 20 h, with prehospital delay alone exceeding 10 h, largely attributable to the absence of organized stroke pathways and limited imaging capacity (Buque et al., 2025). In the Democratic Republic of the Congo, delayed hospital arrival was observed in nearly 90% of patients with acute stroke, with poor stroke symptom recognition and low educational attainment identified as key contributing factors (Kazadi Kabanda et al., 2024). Collectively, these findings place the results of the present study within a broader LMIC context, highlighting that prolonged prehospital delay among older adults is not unique to a single setting but reflects structural and contextual barriers common across health systems with limited resources. These results align with prior research indicating that both clinical and psychosocial factors play a central role in shaping response behaviors to stroke symptoms (Faiz et al., 2019; Kim et al., 2011).

In particular, the negative association between stroke severity and prehospital delay observed in this study is consistent with previous studies demonstrating that more pronounced neurological deficits are more readily recognized as medical emergencies, leading to faster medical attention (Jiang et al., 2019). Conversely, patients experiencing milder symptoms may attribute these manifestations to fatigue, aging, or other benign conditions, resulting in harmful delays in seeking care. This pattern has been consistently reported in LMIC settings, where symptom misinterpretation contributes substantially to prolonged prehospital delay (Damon et al., 2022; Kazadi Kabanda et al., 2024). Such findings are consistent with cognitive appraisal models of illness behavior, which posit that symptom interpretation influences perceived urgency and subsequent help‐seeking actions.

Similarly, the present findings reinforce the importance of stroke knowledge as a modifiable factor in timely care‐seeking behavior. Despite ongoing public health campaigns in Thailand, awareness of stroke warning signs remains insufficient, echoing previous national studies that reported persistent gaps in stroke recognition and response (Sungkarat et al., 2017; Pongpanich et al., 2020). Comparable associations have also been documented in other LMIC contexts, where limited health literacy has been linked to delayed hospital arrival and reduced eligibility for reperfusion therapies (Kazadi Kabanda et al., 2024; Buque et al., 2025). These findings underscore the need for sustained and targeted educational interventions aimed at older adults and their caregivers.

The role of social support further underscores the influence of socio‐cultural factors in shaping prehospital delay in Thailand, where family members and caregivers often guide health‐related decisions. Participants with stronger social networks were more likely to access timely care, paralleling evidence from community‐based studies emphasizing the critical role of caregivers and bystanders in facilitating rapid stroke response. Perceived health status also emerged as a significant factor: individuals who considered themselves healthy were more likely to delay seeking care, possibly due to an underestimation of their vulnerability to stroke and misinterpretation of early symptoms. These findings should be considered within the broader conceptual framework of stroke action awareness. Recent international scholarship highlights that stroke awareness (knowledge of symptoms) is necessary but insufficient to reduce prehospital delay unless it translates into immediate activation of emergency services.

Stroke action awareness frameworks such as Stroke 1‐2‐0, developed for Chinese‐speaking populations using the emergency number 120 as a mnemonic (Zhao & Liu, 2017), and Stroke 911, developed for Spanish‐speaking populations in regions using 911 as the emergency number (Liu et al., 2022), have demonstrated the value of linking symptom recognition directly to emergency action. A randomized comparison in Spanish‐speaking adults found that action‐oriented educational strategies significantly outperformed awareness‐only approaches in improving rapid emergency response (Marquez‐Romero et al., 2025). The present findings suggest that stroke knowledge alone, without action orientation toward activating emergency services (in Thailand, the number 1669), may be insufficient to eliminate prehospital delay. Future interventions in Thailand should therefore integrate action‐oriented stroke education that explicitly links symptom recognition to immediate calling of the national emergency number.

Importantly, these findings highlight that prehospital delay is shaped not only by individual‐level characteristics but also by organizational and system‐level determinants. Consistent with the framework proposed by Botelho et al. (2022), hospital preparedness, efficient triage processes, and coordinated EMSs play a decisive role in determining access to thrombolysis. For Ban Phaeo Hospital, locally applicable improvements include strengthening the existing stroke fast track protocol, enhancing coordination with EMSs, particularly during after‐hours, and exploring scalable solutions such as teleconsultation to support timely clinical decision‐making.

Overall, the present findings emphasize the multifactorial nature of prehospital delay and the need for integrated interventions that address both patient‐related and organizational barriers. Community‐based stroke awareness campaigns, nurse‐led education programs, and health system strengthening initiatives should be tailored to local contexts to improve timely access to acute stroke care. In the context of an aging population, these results further reinforce the pivotal role of gerontological nurses in reducing stroke‐related morbidity through proactive community engagement, targeted health education, and coordinated care across the stroke care continuum.

### 4.1 Organizational Factors and Access to Thrombolysis

Beyond individual‐level determinants, organizational factors play a crucial role in determining access to thrombolytic therapy. Previous research has shown that hospital preparedness, stroke team availability, and prehospital triage systems significantly influence the likelihood of receiving thrombolysis (Botelho et al., 2022). In the Thai context, limitations in after‐hours specialist availability and delays in inter‐hospital transfer may further reduce treatment opportunities. Strengthening stroke protocols, expanding tele‐stroke services, and enhancing EMS coordination are locally applicable strategies to improve thrombolysis rates.

## 5 Limitations

This study has several limitations that should be considered when interpreting the findings. First, the sample was drawn from a single provincial hospital in Thailand, which may limit the generalizability of the results to other regions or more diverse healthcare settings.

Second, the cross‐sectional design restricts the ability to establish causal inference, and subgroup analyses were exploratory, with small sample sizes reducing statistical power. Additionally, potential recall bias may have influenced reporting of symptom onset times. Nevertheless, the consistency of findings with prior literature suggests that the observed associations are robust and relevant. Longitudinal or interventional studies are recommended to validate these associations over time.

Third, several variables, including stroke knowledge, perceived health status, and social support, were assessed using self‐reported instruments, which may be subject to recall bias or social desirability effects.

Lastly, several potentially influential determinants of prehospital delay were not included in this study. These include socioeconomic status, distance from the hospital, transportation access, availability and utilization of EMSs, and psychological factors (e.g., fear, denial, and anxiety). EMS availability and response time in particular are recognized as critical system‐level barriers to timely stroke care, and their absence from the analysis limits the comprehensiveness of the findings. Future studies should incorporate these variables to provide a more complete understanding of the multifactorial drivers of prehospital delay.

Additionally, this study included only stroke survivors, which introduces the potential for survivorship bias. Individuals who experienced very severe strokes and died before hospital admission or before data collection were not represented. As a result, the magnitude of prehospital delay may be underestimated, and the observed associations between clinical and psychosocial factors and prehospital delay may be biased toward less severe cases. This limitation may affect the internal validity of the findings by influencing the strength of observed associations and may also limit external validity, particularly when generalizing the results to the broader population of older adults with ischemic stroke, including those with fatal outcomes.

Future research should incorporate population‐based stroke registries, prehospital mortality data, or prospective designs to provide a more comprehensive understanding of prehospital delay across the full spectrum of stroke severity.

## 6 Implications

The findings of this study carry important implications for clinical practice, public health policy, and nursing education. However, given the cross‐sectional design, these implications are proposed as hypotheses requiring validation through prospective or interventional research rather than as evidence‐based conclusions. First, the significant associations between prehospital delay and stroke knowledge, perceived health status, and social support suggest that targeted educational interventions aimed at older adults and their caregivers may be beneficial. Nurse‐led programs that focus on early recognition of stroke warning signs, risk factors, and the urgency of treatment are hypothesized to reduce delays in care‐seeking behavior; however, this proposition requires confirmation through rigorous interventional studies.

Second, the role of social networks‐including family members and community caregivers‐should be emphasized in public health initiatives. Community‐based strategies that empower caregivers to identify symptoms and assist in emergency response can significantly enhance early hospital arrival rates.

Third, enhancing perceived health awareness among older adults through routine health promotion activities may increase their attentiveness to acute changes and prompt medical action. Nurses, particularly those in community and primary care settings, are well‐positioned to deliver such interventions.

Lastly, the study supports the integration of stroke delay education into broader geriatric care models and health literacy campaigns. Policymakers should consider allocating resources to outreach programs that address both cognitive and contextual barriers to timely stroke treatment. These insights can inform the development of national stroke action plans aimed at reducing disability and mortality among older adults.

Taken together, this study highlighted the multifactorial nature of prehospital delay, integrating both clinical severity and psychosocial determinants. These findings support the need for comprehensive, context‐specific interventions that simultaneously strengthen stroke awareness, empower caregivers, and address individual health perceptions in older adult populations.

## 7 Conclusion

In summary, this study demonstrated that prehospital delay among older adults with ischemic stroke is shaped by an interplay of clinical severity, stroke knowledge, social support, and perceived health status. These findings, while subject to the study's methodological limitations, add to the growing body of evidence that both individual illness representations and socio‐cultural contexts must be considered to effectively address delays in treatment.

Importantly, the results suggest that improving stroke outcomes in older adults requires not only biomedical interventions but also targeted strategies that enhance awareness, leverage caregiver support, and address health perceptions within the community. Future interventions should therefore be multidimensional, culturally informed, and sensitive to the specific needs of aging populations.

## Author Contributions

All listed authors have substantially contributed to the manuscript and have approved the final version: **Harit Sianghwong**: conceptualization, methodology, data curation, investigation, formal analysis, supervision, writing – original draft. **Wipratchaya Thedtong**: conceptualization, methodology, investigation, data curation, validation, formal analysis. **Wanporn Worapornpong**: investigation, project administration. **Pornchai Jullamate**: conceptualization, methodology, supervision. **Chanandchidadussaddee Toonsiri**: validation, methodology, formal analysis.

## Funding

The authors have nothing to report.

## Ethics Statement

This study obtained ethical approval from the Human Research Ethics Board of Ban Phaeo Hospital (Public Organization), **CoA No. 015/68**, **BGH REC No. 013/67** dated February 28, 2025.

## Consent

Written informed consent was obtained from all participants before data collection.

## Conflicts of Interest

The authors declare no conflicts of interest, and no direct or indirect funding body had any role in the study's design or the data's analysis and interpretation.

## Supporting information




**Supplementary Material**: brb371717‐sup‐0001‐SuppMat.docx

## Data Availability

Data available on request due to privacy/ethical restrictions
