# Supplementary material for: Factors Associated With Prehospital Delay Among Older Adults Surviving Ischemic Stroke: A Cross‐Sectional Study
Source: Brain Behav. 2026 Aug 20;16(8):e71717. doi: 10.1002/brb3.71717 (PMC13493700; doi:10.1002/brb3.71717)
Supplement: Supplementary file 1 [file BRB3-16-e71717-s001.docx]

*Supplement: Scatter plots, Histograms, and superimposed normal curves*

*Note. Scatter plots display observed data points (n = 106) for each predictor variable versus prehospital delay (minutes). Solid lines represent ordinary least-squares regression lines; shaded bands indicate 95% confidence intervals. Pearson correlation coefficient (r) and significance level are shown for each panel. Data are illustrative of the reported summary statistics and correlation coefficients.*

*Note. Row 1: Histograms with superimposed normal curves (density scale). Row 2: Q-Q plots with reference line (dashed). Shapiro-Wilk test statistic (W) and p-value are shown in each histogram panel; Q-Q correlation coefficient (r) is shown in each Q-Q panel. Prehospital delay shows marked positive skewness (W = 0.814, p < .001), confirming non-normality and supporting the use of robust sensitivity analyses. The four predictor variables (stroke severity, stroke knowledge, social support, perceived health status) approximate normality (W = 0.955–0.991, p = .001–.698), satisfying the regression assumption of approximately normally distributed residuals*
